# Thermomechanical Investigation of Silicon Wafer Dynamics Within the Melting Regime Driven by Picosecond Laser Pulses for Surface Structuring

**DOI:** 10.3390/ma18245506

**Published:** 2025-12-07

**Authors:** Helen Papadaki, Inam Mirza, Nadezhda M. Bulgakova, Evaggelos Kaselouris, Vasilis Dimitriou

**Affiliations:** 1 Physical Acoustics and Optoacoustics Laboratory, Department of Music Technology and Acoustics, Hellenic Mediterranean University, 74133 Rethymnon, Greece; ddk134@edu.hmu.gr (H.P.); vagfem@hmu.gr (E.K.); 2 Institute of Plasma Physics and Lasers-IPPL, University Research and Innovation Centre, Hellenic Mediterranean University, 74150 Rethymnon, Greece; 3 FZU—Institute of Physics of the Czech Academy of Sciences, Na Slovance 1999/2, 182 00 Prague, Czech Republic; mirza@fzu.cz (I.M.); nadezhda.bulgakova@fzu.cz (N.M.B.)

**Keywords:** finite element analysis, Si wafers, LIPSSs formation, laser–matter interaction

## Abstract

Laser-induced periodic surface structures (LIPSSs) on silicon, generated by ultrashort pulsed lasers, provide an efficient means to tailor surface functionality. This work presents a multiphysics finite element study on the thermomechanical dynamics of silicon wafers irradiated by picosecond laser pulses, focusing on the melting regime where thermomechanical and hydrodynamic effects dominate. To illustrate the sequential nature of laser scanning, single-pulse irradiation models are developed as thermomechanical analogues of sequential laser irradiations. By positioning the laser focus near reflective boundaries and corners of the target, these models reproduce the stress wave interference that would occur between successive pulses in laser scanning. The results show that periodic surface structures are enhanced from mechanical standing wave interference within the molten layer, forming ripples with near-wavelength periodicity. The penetration depth (PD) is identified as a key factor controlling the duration and stability of these ripples: shallow PDs (75–150 nm) yield distinct, persistent patterns, while deeper PDs (~2.5 μm) lead to extended melting and hydrodynamic smoothing. Simulations of sequential laser pulse irradiations confirm that residual stresses and strains from the first pulse amplify deformation during the second, enhancing ripple amplitude and uniformity. Thus, the role of controlled excitation of mechanical standing waves governed by PD, boundary geometry, and pulse sequencing, in deterministic LIPSSs formation on silicon is revealed.

## 1. Introduction

Laser-induced periodic surface structures (LIPSSs) are quasi-periodic micro- or nanostructures that form on a material’s surface upon irradiation with laser light, particularly from ultrashort pulsed lasers [1,2,3,4,5]. They consist of repetitive ripple-like patterns with a periodicity typically close to the laser light’s wavelength. LIPSSs can be generated on a wide variety of materials, including metals, semiconductors, and polymers [3,6,7,8,9,10]. The formation of ripples on the material’s surface alters the properties of the affected area, including its roughness, wetting behavior, and optical absorption coefficient. The induction of LIPSSs is explained by combining two fundamental approximations, the interference of surface electromagnetic waves (SEWs) and the matter reorganization. According to the first approach, the incoming laser pulse, the SEWs generated within the irradiated spot, and the SEWs scattered from the crater edge formed by previous pulses or due to surface roughness interfere, developing a local energy modulation that is imprinted onto the material via absorption [11,12,13]. The second approach attributes LIPSSs formation significantly to hydrodynamic flow during the material’s melting phase and matter reorganization during re-solidification [11,12,14]. The current work presents a novel thermomechanical study on periodic surface structuring of Si wafers by laser-induced mechanical waves not including the contribution of electromagnetic interference.

**Figure 1:**
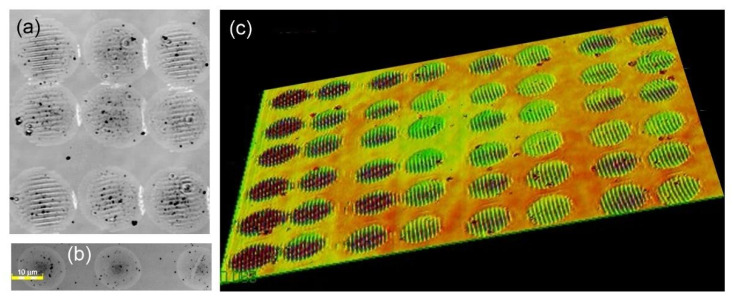
(**a**) Optical image of an array of irradiation spots with 1/e^2^ diameter of 25 µm and the distance between spot centers of ~16 µm. Note that the precision of the galvo scanner (about ±2.5 μm on the sample surface) did not allow for a perfect spacing between the spots [28]. (**b**) Typical image of the irradiation spots when the distance between spot centers was increased to 25 µm with absence of LIPSS signs. (**c**) A slanted view of the LIPSS array obtained under the conditions of (**a**). All three images have been obtained at laser fluence of 1.04 J/cm^2^.

The formation of LIPSSs driven by ultrashort pulsed laser irradiation presents a promising and efficient method for tailoring surface properties. While LIPSS formation has been extensively studied with femtosecond (fs) pulsed lasers [15,16,17,18,19], the role of picosecond (ps) pulsed lasers in this process lacks systematic investigations [20,21]. Laser pulses of ps duration offer a unique thermal regime since they are long enough to allow for electron-lattice coupling and melting and yet short enough to minimize severe thermal damage, allowing a fascinating interplay between photophysical and photothermal processes that dictates the final surface morphology. It is particularly significant to investigate the influence of laser parameters, pulse overlap, material properties, and the boundaries that reflect mechanical waves on LIPSS induction. This is especially critical for Si wafers, given their extensive use in technological devices [22,23]. In the research work presented in [24], we observed that regular LIPSSs can be produced on Si surfaces upon ps laser scanning at rather small overlapping between irradiation spots via experiments which are representatively depicted in Figure 1, and corresponding simulations validated the results. Specifically, scattering from the laser-modified regions considering the hydrodynamic effects was successfully simulated, linking the LIPSS formation with the modification fingerprints induced by each successive laser pulse. Furthermore, the experimental campaign performed on laser irradiation of Si targets using infrared (1064 nm) ps pulsed laser with a Gaussian spatial beam profile, effective focal spot diameter (2 *w_o_*) of laser on the target surface of ~25 μm, and peak fluence on target surface of ~2 J/cm^2^ led to findings of equivalent importance. The post-processing of the irradiated Si samples determined that LIPSSs within adjacent irradiation spots start to appear at about 19–20 mm of laser focal spot centre distances, and the amplitude of the ripples starts to increase and become more prominent with reduced inter spot distance [24]. It should be noted that similar experiments performed for molybdenum and titanium samples did not demonstrate such a replication effect. This can relate to the difference in the quality factors Q of these materials [25,26] among which Si exhibits an extraordinary Q factor at enhanced temperatures [27] thus, low-dissipating acoustic waves in Si, via their interference, can contribute to the LIPSS formation.

Numerical simulations represent a powerful methodology for investigating laser-matter interactions. By employing appropriate laser parameters and material properties, these simulations can accurately predict resultant structural and property modifications. Extensive research has utilized numerical models to elucidate the behavior of solids under laser irradiation, with a significant focus on silicon (Si) wafers subjected to ultrashort laser pulses [28,29,30]. In our previous works [31,32,33], we developed and validated three-dimensional (3D) multiphysics finite element method (FEM) models, integrating coupled thermo-structural physics, to describe the response of thin metal films and Si targets exposed to nanosecond (ns) laser pulse irradiation. To describe the behavior of the Si targets, a suitable material model is adopted, accounting for elastoplastic effects and phase change induced by the high temperatures generated during irradiation. The hydrodynamic and bulk response of the material is modeled using a proper equation of state. The low values of density, thermal expansion coefficient, and absorption coefficient, significantly influence the thermomechanical response of the irradiated Si in contrast to the dense metals commonly studied in the literature [31,32]. To enable the detailed representation of surface modifications during and after the laser-target interaction presented in [24,33], the 3D FEM model was discretized by a highly refined uniform mesh to accurately capture the dynamic response of the solid Si target.

Aiming to decode the dynamic effects of sequential laser pulse irradiation across timescales from ps to ms, we systematically examine the thermomechanical equivalent of a single-pulse irradiation positioned near the peripheral boundaries of the rectangular solid Si target. The reflective plane boundaries may react like the boundaries induced from the leading successive pulse in the target, during laser scanning. Therefore, the boundary conditions of the FEM model, at the four peripheral plane boundaries of the solid Si target, are here set to free, unlike the models that simulated the interactions in our previous research works. Thus, we let matter at these four boundary planes to react freely and reflect the mechanical waves, in the same way that material modifications (“fingerprints”) induced by successive laser pulses do during laser scanning at any top-surface point on the target. These free boundaries effectively model the permanent material modifications induced by successive laser pulses, providing critical insight into subsequent LIPSS formation. Based on the optimal focal spot separation for LIPSS induction established in [24], we position the laser spot adjacent to a single boundary plane at the right of the target, to simulate simple wave interference. Additionally, since the successive leading laser pulse modification induces boundary fingerprints of circular/ellipsoidal geometrical forms, we simulate the irradiation equidistant from two adjacent boundary planes, at the top-right target vertex, to similarly generate and study complex wave patterns. These two thermomechanical configurations—equivalent to sequential irradiation—simulate how material modifications from a leading pulse precondition the surface and govern the mechanical wave dynamics of a subsequent pulse in both time and space. The results establish the controlled excitation of mechanical waves as an important mechanism for LIPSS formation, providing a pathway, which in synergy with the interference of SEWs, allows for deterministic laser-based surface patterning.

## 2. Numerical Modeling and Simulation

The solid square Si target FEM model is discretized by a highly refined uniform mesh to accurately capture its dynamic response while and after interacting with Gaussian-profile ps laser pulses and solved in Ls-DYNA R15.0.2 [34]. The multiphysics coupled thermal–mechanical FEM models are simulated on the High-Performance Computer (HPC) Advanced Research Information System (ARIS) [35].

### 2.1. Mathematical Description

Given the ultrashort nature of the laser pulse, convective and radiative losses are negligible thus laser-silicon interaction is governed by heat conduction. The one-temperature heat conduction equation is:

(1) $$\rho \left(r,T\right)C_{p}\left(r,T\right)\frac{\partial T(r,t)}{\partial t}-\nabla [k\left(r,T\right)\nabla T\left(r,t\right)]=Q\left(r,t\right)$$

where ***r*** is the vector of location for cartesian coordinates *x*, *y*, *z*, *Τ* is the temperature, *ρ* is the mass density, *C_p_*, *k* are the temperature-dependent specific heat at constant pressure and thermal conductivity, respectively, while *Q*(***r***,*t*) is the absorbed laser energy per unit volume, per unit time by the sample. The latent heat of melting is also considered when temperature exceeds the melting point. As previously mentioned, the ps pulse has a Gaussian spatiotemporal profile. Consequently, the absorbed volumetric heat flux, *Q*(***r***,*t*), is given by the following equation:

(2) $$Q(x,y,z,t)=I_{0}(t)(1-R){a_{b}e}^{-4ln2(\frac{t^{2}-t_{0}^{2}}{t_{0}^{2}})}e^{-(\frac{x^{2}+y^{2}}{r_{0}^{2}})}e^{-a_{b}z}$$

where *I*_0_(*t*) is the temporal profile of the laser intensity, and *r_0_* and *t_0_* are the beam radius (at 1/e^2^ intensity level) and the FWHM laser pulse duration, respectively. The term (1 − *R*) *I*_0_(*t*) represents the portion of the laser energy that propagates into the target, where *R* is the surface reflectivity. The coefficient *α_b_* is the absorption coefficient, which depends on temperature, laser wavelength, surface polish, and the doping concentration of the sample. The exponential term exp(−*α_b_z*) describes the attenuation of the laser intensity with depth *z*.

Rapid local heating and associated thermal expansion generate a stress field, resulting in ultrasonic waves that propagate in the material. The equation of wave propagation is written in the form:

(3) $$\rho \left(r,T\right)\frac{\partial^{2}U(r,t)}{\partial t^{2}}=\mu \nabla^{2}U\left(r,T\right)+\left(\lambda +\mu \right)\nabla \left[\nabla U\left(r,t\right)\right]-a\left(3\lambda +2\mu \right)\nabla T(r,t)$$

where *U* represents the displacement, *α* the thermal expansion coefficient and *λ*, *μ* are Lame constants depending on the material. The mechanical behavior of the Si target can be expressed by the following Equations (4) and (5) where *σ_ij_* and *ε_ij_* are the stress and strain tensors in the *ij* plane, respectively, and *T*_0_ the ambient temperature:

(4) $$\sigma_{ij}=2\mu \varepsilon_{ij}+\lambda \varepsilon_{kk}\delta_{ij}-\left(3\lambda +2\mu \right)a(T-T_{0})\delta_{ij}$$

(5) $$\varepsilon_{ij}=\frac{1}{2}\left(\frac{\partial U_{i}}{\partial x_{j}}+\frac{\partial U_{j}}{\partial x_{i}}\right)$$

The hydrodynamic and bulk behavior of the solid target is described using the Grüneisen equation of state which gives the pressure as function of volume and internal energy of the material. Equation (6) describes the compressed materials and (7) the expanding materials:

(6) $$p=\frac{{\rho_{0}C}^{2}\mu_{d}[1+\left(1-\frac{\gamma_{0}}{2}\right)\mu_{d}-\frac{\alpha_{1}}{2}{\mu_{d}}^{2}]}{{\left[1-\left(s_{1}-1\right)\mu_{d}-s_{2}\frac{{\mu_{d}}^{2}}{\mu_{d}+1}-s_{3}\frac{{\mu_{d}}^{3}}{{\left(\mu_{d}+1\right)}^{2}}\right]}^{2}}+\left(\gamma_{0}+\alpha_{1}\mu_{d}\right)E$$

(7) $$p=\rho_{0}C^{2}\mu_{d}+\left(\gamma_{0}+\alpha_{1}\mu_{d}\right)E$$

In these equations *C* represents the sound speed, *E* the internal energy per initial volume and *γ*_0_, *α*_1_, *s*_1_, *s*_2_, *s*_3_ are unitless constants. *s*_1_, *s*_2_, and *s*_3_ are the coefficients of the slope in the *u_p_*–*u_s_* curve (the shock wave velocity *u_s_* varies linearly, with respect to the particle velocity *u_p_*), *γ*_0_ is the Grüneisen parameter and *α*_1_ represents the first order volume correction to *γ*_0_. The term *μ_d_* is a volumetric parameter defined by the relation *μ_d_ =* (*ρ*/*ρ*_0_) − 1.

### 2.2. Geometry, Discretization & Simulation Parameters

The 3D FEM model, capable of simulating the response of a Si target under ps laser irradiation, was originally developed and validated for the study of solid Si behavior under ns and ps laser irradiation in [33] and in [24], respectively. Herein, the 3D multiphysics model is adapted to investigate the Si response under single- and sequential- ps laser irradiations, varying in space and in time. In the cases of simulating single pulse irradiations next to the target boundaries the boundary conditions of the four peripheral plane boundary surfaces are modified and set to free.

The rectangular workpiece with dimensions of 160 µm × 160 µm × 3 µm (along the X, Y, and Z axes, respectively) is discretized using eight-node hexahedral solid elements of dimensions 1 µm × 1 µm × 75 nm. The simulation region consists of 1,024,000 elements. To ensure both numerical accuracy and computational efficiency, a mesh size of 1 μm × 1 μm × 75 nm was selected after an extensive mesh-sensitivity study. Several alternative element dimensions—both larger (3 μm × 3 μm × 150 nm, 2 μm × 2 μm × 75 nm) and smaller (1 μm × 1 μm × 50 nm, 0.5 μm × 0.5 μm × 50 nm)—were evaluated. The chosen configuration was found to accurately resolve the steep thermal and stress gradients while capturing lateral wave propagation with negligible numerical errors. Further refinement yielded negligible improvements in the predicted temperature and displacement fields, whereas coarser meshes led to noticeable loss of resolution. Thus, the final element size provided an optimal balance between solution fidelity and computational cost, particularly given the long simulation times required (up to hundreds of nanoseconds or microseconds). This configuration was found to be suitable for describing the thermo-mechanical response of the target across both short (ps) and long (ms) spatiotemporal scales [24,33].

**Figure 2:**
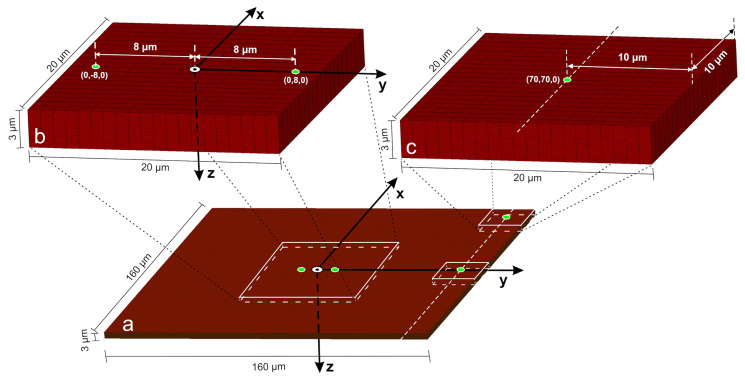
(**a**) Discretized Si model geometry, irradiated on the top surface (xy-plane). (**b**,**c**) Zoomed details. (**a**) shows the discretized Si model geometry, irradiated on the top surface (xy-plane). The two green spots on either side of the center, positioned 8 μm from the target’s origin, mark the centers of two sequential laser pulses with a 25 μm diameter focal spot. The first pulse is focused on point (0, 8, 0), as shown in the zoomed detail of (**b**), while the second pulse is focused at (0, −8, 0). The second pulse irradiates the target after a minimum delay of 20 μs, ensuring the target has cooled to ambient temperature. In this sequential-pulse model, non-reflective (outflow) boundary conditions are applied to the non-irradiated surfaces to prevent wave reflections and model a semi-infinite domain. This contrasts with the single-pulse models, where the laser is positioned near the sample’s natural free boundary surface planes. These four free surfaces (stress-free boundaries) inherently reflect stress waves, and their strategic placement is used to physically simulate the wave-reflecting fingerprints, created by successive pulses in a scanning process. The imposition of the free boundary conditions in the single-pulse model cases is the main difference in our simulation model with our previous research works.

To decipher the complex sequential-pulse phenomena reported in [24], we employ a thermomechanical analog single-pulse model. The study in [24] established that a laser spot center distance below 25 μm is required for LIPSS formation and stress/strain amplification. Accordingly, we simulate a single ps laser pulse focused on point (0, 70, 0), placing it 10 μm from the right plane peripheral boundary, as shown in Figure 2a. To further investigate the effect of a circular/ellipsoidal geometrical form modification boundary from a prior pulse during laser scanning, a second single-pulse simulation is performed, focusing the laser at the upper-right vertex, point (70, 70, 0), 10 μm from both adjacent boundary planes (see zoomed detail in Figure 2c).

The thermomechanical simulations are performed by the implicit–explicit staggered coupling scheme of the LS-DYNA solver. The heat conduction equation is solved with the implicit thermal solver, while the mechanical response is integrated using the explicit central-difference solver to accurately capture high-frequency stress waves. At each time step, the updated temperature field obtained from the implicit thermal solution is transferred to the explicit mechanical solver to apply thermal strains and update temperature-dependent material properties.

### 2.3. Material Properties and Laser Parameters

The Johnson-Cook (J-C) [36] material model is employed to describe the mechanical response of Si under the extreme conditions of ps laser irradiation. This choice is justified by the well-documented brittle-to-ductile transition in Si at high temperatures and strain rates. Under the intense, localized heating of the laser pulse, Si ceases to behave as a brittle solid and enters a softened, plastically deformable state. The J-C model serves as a robust phenomenological framework to capture this effective irreversible deformation and the resulting stress–strain response in the melted and thermally softened regions, consistent with its application in other studies of Si under high thermo-mechanical loading [37,38]. The J-C material model provides the flow stress *σ* (Equation (8)) and in case of high strain rates includes a fracture model that defines the equivalent plastic strain *ε_f_* in case of damage (Equations (9) and (10)):

(8) $$\sigma =(A+B\varepsilon^{n})\left(1+Cln\frac{\dot{\varepsilon }}{\dot{\varepsilon_{0}}}\right)\left(1-(\frac{T-T_{r}}{T_{m}-T_{r}}{)}^{m}\right)$$

(9) $$\varepsilon_{f}=\left(D_{1}+D_{2}e^{D_{3}\frac{p}{\sigma_{VM}}}\right)\left(1+\frac{D_{4}ln\dot{\varepsilon }}{\dot{\varepsilon_{0}}}\right)\left(1+D_{5}\frac{T-T_{r}}{T_{m}-T_{r}}\right)$$

(10) $$D=\Sigma \left(\frac{\Delta \varepsilon }{\varepsilon_{f}}\right)$$

In Equations (8)–(10) *T* represents the temperature of the target, *T_r_* the room ambient temperature and *T_m_* the melting point of the material. Parameters *A*, *B*, *C*, *n*, *m* are experimental constants which depend on the material, *p* is the pressure, while $\dot{\varepsilon }$ and $\dot{\varepsilon }$_0_ are the strain rate and the reference strain rate, respectively, and *D*_1_–*D*_5_ are the failure parameters of the material. Finally, *D* is the damage parameter. When *D* becomes 1, the material fractures. The temperature dependent values of the mechanical properties and thermal properties, as well as the J-C parameters of the Si sample used in the simulations can be found in the supplementary data of [28].

The beam diameter on the sample surface is assumed to be 25 μm (at 1/e^2^ intensity level). In all simulations, the FWHM pulse duration was 6 ps. The reflectivity of Si at 1030 nm is taken for simplicity to be 0.32 at room temperature [39], and the absorption coefficient is temperature dependent [40]. A laser fluence of 1.64 J/cm^2^ is considered to study the occurring physical phenomena in the melting regime.

The material properties of Si wafers vary depending on dopant concentration, crystal quality, the type of irradiated surface, and rising temperature. The simulations revealed that a critical factor influencing the results was the laser light’s penetration depth. In pure Si crystals, the penetration depth can be calculated as 1/α_b_, where α_b_ is the absorption coefficient for a specific wavelength. However, in Si wafers, the absorption coefficient depends on the proportion of dopants, particularly in the case of highly doped wafers [41,42,43]. More significantly, it increases substantially with rising temperature. At elevated temperatures, the absorption coefficient can increase dramatically [40,44], making it challenging to accurately calculate the penetration depth, which becomes smaller than the conventional estimate of 1/α_b_ [45]. For a very high dopant Si wafer layered material, the penetration depth can be less than 1 μm for IR laser light [46,47]. For this reason, different mean values of penetration depths were simulated.

## 3. Results

A single-temperature model describes the laser-matter interaction, justified by silicon’s characteristic electron-lattice thermalization time of ~10 ps [29,48], which is comparable to the pulse duration. To investigate how the boundary geometry of a leading pulse influences subsequent LIPSS formation, we employ two equivalent single-pulse irradiation models that probe sequential thermomechanical effects: one with the pulse focused near a reflective plane boundary, and another near a reflective corner vertex. Three distinct irradiation scenarios are examined: (i) a ps laser pulse applied 70 μm from the target’s center and 10 μm from its right edge; (ii) a pulse focused 10 μm from both adjacent edges at a corner; (iii) two successive, partially overlapping pulses to demonstrate how material modifications from the leading pulse affect LIPSS formation by the second pulse.

These simulations are performed for penetration depth (PD) of 75 nm, 150 nm, and 2.5 μm, revealing this parameter’s crucial role in LIPSS formation, which depends on material properties and sample characteristics. The duration of the molten state within the laser spot is brief, lasting only 1–5 ns depending on the penetration depth. In contrast, the subsequent cooling of the entire target to ambient temperature occurs over a significantly longer period. For single pulses, the total simulation times were approximately 10 μs (75 nm PD), 20 μs (150 nm PD), and 50 μs (2.5 μm PD) for the target to return to ambient temperature. For sequential pulses, simulation times roughly doubled, except for the 2.5 μm PD case, which required ~500 μs after the second pulse for cooling down.

### 3.1. Single-Pulse Irradiation Next to a Reflective Boundary Plane

**Figure 3:**
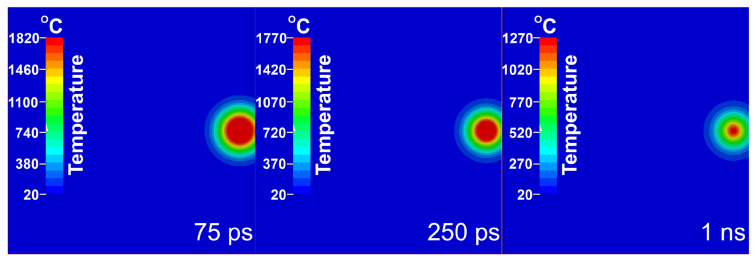
Evolution of temperature distribution for PD 75 nm.

The thermal response of the Si target varies significantly with PD. For a PD of 75 nm, the surface temperature peaks at 1800 °C at 20 ps and remains above silicon’s melting point (1414 °C) for ~900 ps. The heated region is superficial, with temperatures exceeding 1400 °C in a 22 μm diameter area and higher than 1000 °C confined to a depth of ~150 nm. Consequently, the target cools to ambient temperature within ~9 µs. For a PD of 150 nm, the maximum surface temperature reaches 1430 °C at 20 ps and remains above melting for ~3 ns. A smaller 18 μm diameter region exceeds 1400 °C, but the heat penetrates more deeply, with temperatures higher than 1000 °C reaching ~200 nm. The temperature at a 3 μm depth is 160 °C, compared to 90 °C for the 75 nm PD case. The deeper heating results in a longer cooling time of ~20 μs to ambient temperature. For a PD of 2.5 μm, the surface temperature peaks at 1420 °C at 22 ps, sustaining a molten state for ~100 ns. A 20 μm diameter region exceeds 1400 °C, with temperatures higher than 1300 °C extending to a depth of 2.2 μm. The temperature at 3 μm depth exceeds 1200 °C. As the entire target is heated, it requires ~0.2 ms to reach a uniform thermal equilibrium at 55 °C. The evolution of temperature distribution for PD 75 nm is presented in Figure 3 for three representative temporal moments.

The thermal response of the Si target varies significantly with PD, yet the surface peak temperature remains near the melting point (~1420–1430 °C) for PDs of 150 nm and 2.5 µm. This occurs because the melting process itself acts as a temperature-regulating mechanism; once the melting point is reached, absorbed energy is primarily channeled into the latent heat of fusion, preventing a significant further temperature increase. The key difference lies in the melt’s characteristics: a shallow PD (75–150 nm) creates a thin, short-lived molten layer (~150–200 nm deep, lasting ~1–3 ns), leading to rapid cooling (µs timescale). In contrast, a deep PD (2.5 µm) generates an extensive molten volume (extending 2.2 µm deep, lasting ~100 ns), resulting in a much longer cooling time (~0.2 ms). Thus, while the peak temperature is regulated by the phase change, the PD governs the spatial and temporal extent of the melt, which critically influences the resulting surface morphology.

**Figure 4:**
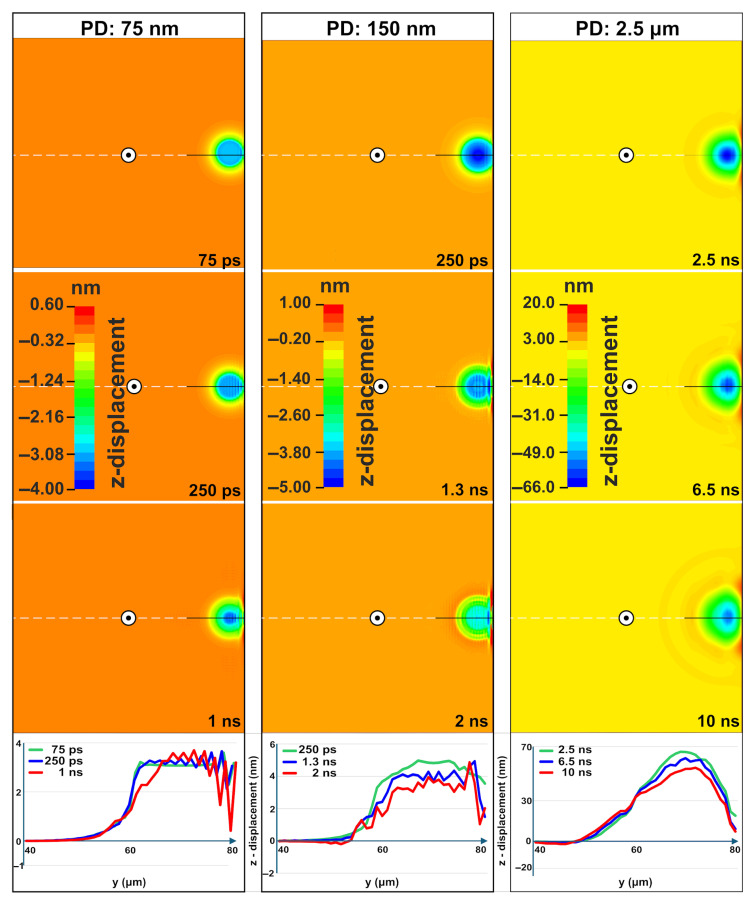
Contour plots for the evolution of the vertical z-displacement for three different PDs and the graphs of the corresponding lineouts.

Figure 4 presents the evolution of vertical z-displacement following laser irradiation at three different penetration depths. The observed surface ripples are primarily driven by a thermomechanical mechanism where mechanical standing waves form within the molten layer. The rapid, localized heating launches a broadband thermoelastic wavepacket into the surrounding material while simultaneously driving a strong transient surface bulge. As the heated region relaxes and the molten geometry becomes quasi-static, the earliest wave components reaching the nearby free boundary generate reflected waves. The interaction between the continuing wavepacket and these returning components gradually establishes a standing wave pattern within the molten layer. The standing wave establishes regions of compression and rarefaction within the molten Si itself, and the resulting pressure gradients displace the liquid surface hydrodynamically, creating ripples that become permanently imprinted upon re-solidification. The periodicity of these ripples (approximately 1.5–2 µm) emerges from the acoustic properties of Si and the boundary geometry, naturally occurring on the same order of magnitude as the laser wavelength (1.03 μm) due to the micrometer-scale confinement of the energy deposition.

For a PD of 75 nm, the interference pattern becomes clearly visible by 250 ps with a periodicity close to the laser wavelength. The amplitude of the pattern increases by 1 ns, and the deformation depth stabilizes within 7.5 µs. At 10 µs, the maximum resolidified deformation is 35 nm, with distinct ripples remaining visible. For a PD of 150 nm, interference patterns emerge by 250 ps and are fully formed by 1.3 ns. The amplitude starts to reduce after 2 ns due to energy dissipation. At 20 µs, the maximum resolidified deformation depth is 95 nm, with faint persisting ripples. For PD 2.5 µm, higher-amplitude standing waves form ripples by 2.5 ns. However, these incipient features are weakened by hydrodynamic flow within the extensive and long-lived (~100 ns) molten pool. Moreover at 10 ns an ultrasonic wave is also evident and in accordance with the detailed study published in [33]. The maximum resolidified deformation depth is 450 nm, achieved when the target reaches thermodynamic equilibrium at 100 µs.

**Figure 5:**
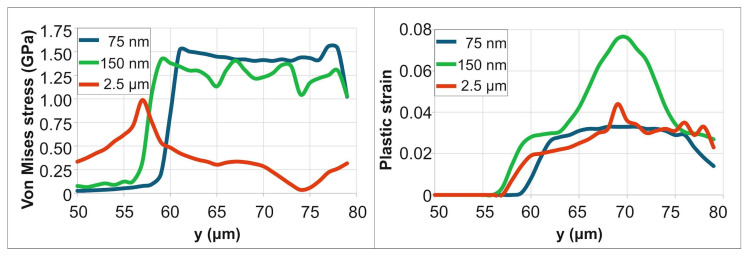
Residual stress and plastic strain distributions for varying PDs.

For a PD of 75 nm, the Von Mises stress peaked at approximately 1.7 GPa at 25 ps, dropped to 0.8 GPa by 500 ps, and subsequently increased to a final value of 1.6 GPa (end of simulation time). For a PD of 150 nm, the stress fluctuated between 1.1 GPa and 1.8 GPa before stabilizing at a final value of 1.6 GPa by 10 μs. For a PD of 2.5 μm, the stress varied between 1.1 GPa and 1.8 GPa over a longer duration (~50 μs), ultimately stabilizing at a lower residual stress of 1 GPa. Figure 5 demonstrates the spatial distribution of the residual stresses and strains, in the irradiation region, at the end of simulation time (10 μs for 75 nm PD, 20 μs for 150 nm PD and 50 μs for 2.5 μm PD. The von Mises stress results indicate that for lower PD (75 nm and 150 nm), high residual stress is intensely concentrated at the boundary of the laser spot. In contrast, the deeper penetration depth (2.5 μm) creates a broader zone of material modification with a lower peak stress. For a PD of 75 nm, the residual plastic strain within a central 10 µm diameter area is approximately 0.027–0.033. For a PD of 150 nm, the strain is significantly higher (0.06–0.076) but confined to a smaller central region of 5 μm diameter. For a PD of 2.5 μm, a substantial plastic strain (0.02–0.04) is sustained across the entire irradiated zone.

### 3.2. Single-Pulse Irradiation Next to a Reflective Boundary Vertex

**Figure 6:**
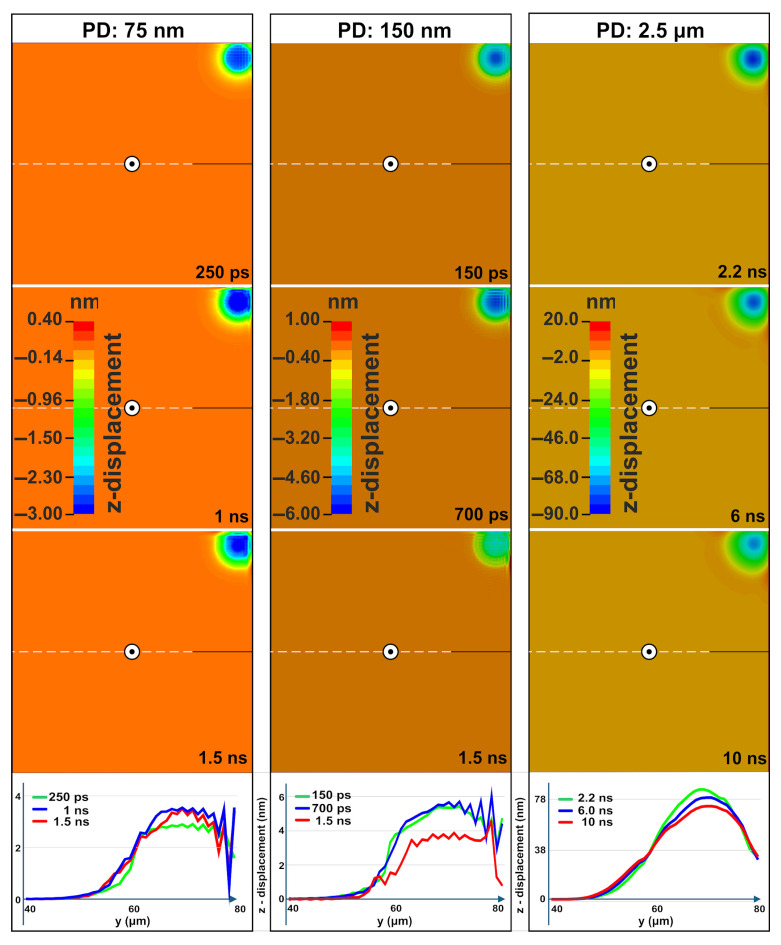
Contour plots for the evolution of the vertical z-displacement for three different PDs and the graphs of the corresponding lineouts.

The irradiation close to the upper right boundary vertex of the sample (see Figure 2c) consistently yields measurably lower maximum temperature, von Mises stress, and residual plastic strain compared to the single-pulse irradiation case next to a reflective boundary Plane. For instance, at a PD of 75 nm, the maximum deformation depth was reduced from 35 nm (straight edge) to 30 nm (corner), a ~15% decrease. Figure 6 presents the evolution of vertical displacement for irradiation near a corner at three different PDs. For a PD of 75 nm, well-defined periodic structures are established by 250 ps, with the amplitude peaking around 1 ns before beginning to decay after 1.5 ns due to energy dissipation. For a PD of 150 nm, wave patterns begin to emerge by ~250 ps. The amplitude increases significantly by 700 ps but is substantially reduced by 1.5 ns as dissipation occurs within the thinner molten layer. For a PD of 2.5 μm, higher-amplitude standing waves form ripples by 2.2 ns but are subsequently weakened by hydrodynamic flow in the extensive molten pool—behavior consistent with the straight-edge case. Although acoustic interference patterns re-emerged transiently during solidification, they ultimately failed to stabilize into permanent surface structures.

Critically, vertical displacements are consistently smaller during the irradiation next to the vertex. The doubly reflected waves, from both reflective vertical boundary planes, interfere in a manner that reduces out-of-plane motion while minimally affecting longitudinal displacements. This results in lower final displacement magnitudes of 30 nm, 40 nm, and 230 nm for penetration depths of 75 nm, 150 nm, and 2.5 μm, respectively.

**Figure 7:**
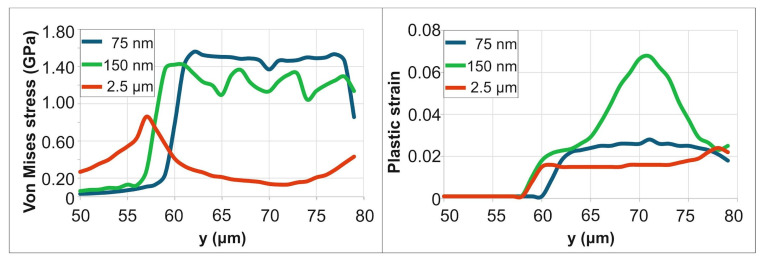
Residual stress and plastic strain distributions for varying PDs.

Figure 7 demonstrates the spatial distribution of residual stresses and strains within the irradiation region near the corner, at the end of simulation time (10 μs for 75 nm PD, 20 μs for 150 nm PD and 50 μs for 2.5 μm PD). Consistent with the earlier observation, this configuration exhibits lower residual stresses and strains. As shown in Figure 7, the peak von Mises stress for a 75 nm PD near the corner is approximately 1.6 GPa, compared to ~1.7 GPa for the straight-edge case (Figure 5). The von Mises stress results align with the findings from Section 3.1: for shallow penetration depths (75 nm and 150 nm), high residual stress is intensely concentrated at the boundary of the laser spot. In contrast, the deeper PD (2.5 μm) creates a broader zone of material modification with a lower peak stress. In contrast, the deeper PD (2.5 μm) creates a broader zone of material modification with a lower peak stress. For a PD of 75 nm, the residual plastic strain within a central 10 µm diameter area is approximately 0.023–0.03. For a PD of 150 nm, the strain is significantly higher (0.055–0.07) but confined to a smaller central region of 5 μm diameter. For a PD of 2.5 μm, lower plastic strains (0.015–0.018) are observed in the irradiated area.

Based on the results of Section 3.1 and Section 3.2, we conclude that for cases with a smaller PD a higher maximum temperature, a shorter cooling time, and reduced vertical displacements are achieved. While ripple formation is initiated for all cases, a deep molten pool at the 2.5 μm PD delays their clear formation. Subsequently, these ripples are depleted by extensive hydrodynamic flow as the material reorganizes. PD influences ripple dynamics only through its effect on the thermal absorption profile, not as a direct mechanical parameter.

The observed timescales for ripple formation—ranging from ps for shallow PDs to ns for deeper PDs—are a direct physical consequence of the PD-governed thermal response. A shallow PD (e.g., 75–150 nm) generates a thin, transient molten layer, requiring standing waves to form and imprint the surface within a short (~1–3 ns) time window. In contrast, a deep PD (e.g., 2.5 µm) creates a large, long-lived molten pool where hydrodynamic flow delays the emergence of stable ripples, shifting their clear formation to the nanosecond scale. Therefore, the variation in formation time is a predictable outcome of how the PD controls melt duration and hydrodynamic stability.

### 3.3. Sequential Laser Pulses Irradiation

To investigate the influence of the material modifications induced from the leading pulses on the following, we simulated the irradiation sequence of two pulses, by focusing the second laser pulse at the distances of 24, 20, 16 and 12 μm on the y-axis, from the first focal spot center, after targets’ cool down to the ambient temperature. The results established a separation threshold: for distances greater than 20 μm, the first pulse had a negligible effect. Conversely, a 12 μm separation resulted in excessive overlap, obscuring individual pulse effects. Consequently, the 16 μm separation distance is further analyzed as a representative case that exhibits clear thermomechanical interaction without significant spatial overlap. These findings validate the experimental observations in [24] and confirm that the 10 μm distance—strategically chosen for our single-pulse analogues based on those experiments—accurately captures the critical interaction distance for constructive wave interference.

For a PD of 75 nm, the first pulse induced a peak temperature of 1805 °C at 18 ps, which was sustained until 100 ps. The shallow penetration depth confined most thermal energy to a depth of approximately 150 nm. The material within a central 10 µm diameter region remained above the melting point for ~1 ns. The thermomechanical response produced a maximum von Mises stress of approximately 1.6 GPa at 25 ps, and a plastic strain of 0.033 on the top surface. A second pulse was applied 10 µs later, after the target cooled to ambient temperature, with its focal point offset by 16 µm. This pulse produced nearly identical thermal behavior, reaching a similar peak temperature of 1800 °C. As in the single-pulse case, the target cooled to ambient temperature within ~10 µs.

**Figure 8:**
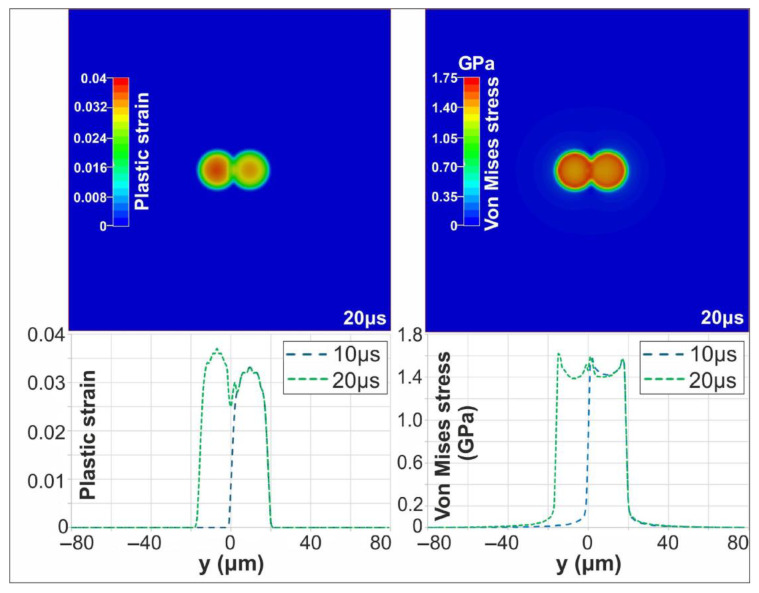
Contour plots of plastic strain and Von Mises stress at 20 μs (**top**); Evolution of plastic strain and Von Mises stress following the first and second pulses (**bottom**).

The mechanical response, however, was significantly more pronounced. The second irradiated area experienced higher stress, with maximum plastic strain and von Mises stress reaching 0.037 and 1.62 GPa, respectively, exceeding the first pulse’s values. Figure 8 shows the evolution of von Mises stress and plastic strain distribution following the first and second pulses. Once the target has cooled to ambient temperature (10 μs after the second pulse), the effect of the successive, partially overlapping pulses remains more pronounced, as evidenced by the higher residual stress and strain fields. Furthermore, the maximum deformation depth was ~38 nm after the first pulse and increased to 55 nm following the second pulse.

**Figure 9:**
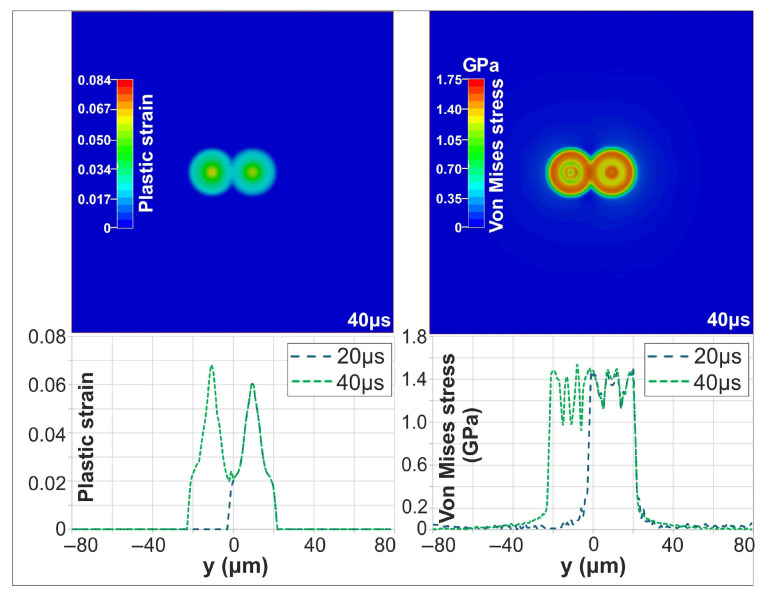
Contour plots of plastic strain and Von Mises stress at 40 μs (**top**); Evolution of plastic strain and Von Mises stress following the first and second pulses (**bottom**).

For a PD of 150 nm, the first pulse produced a peak temperature of 1430 °C at 18 ps. The second pulse resulted in nearly identical thermal behavior, reaching the same maximum temperature and heated depth. The mechanical response, however, was significantly more pronounced, indicating an increased sensitivity to the material and surface properties modified by the first pulse. During the second pulse, the maximum von Mises stress and plastic strain increased to approximately 1.54 GPa and 0.068, respectively, exceeding the first-pulse values of 1.48 GPa and 0.06. As in the single-pulse case, the target cooled to ambient temperature within approximately 20 µs. Figure 9 shows the evolution of the Von Mises stress and plastic strain distribution following the first and second pulses, once the target has cooled to ambient temperature (20 μs after the second pulse). Furthermore, the maximum deformation depth was ~90 nm after the first pulse and increased to 110 nm following the second pulse.

**Figure 10:**
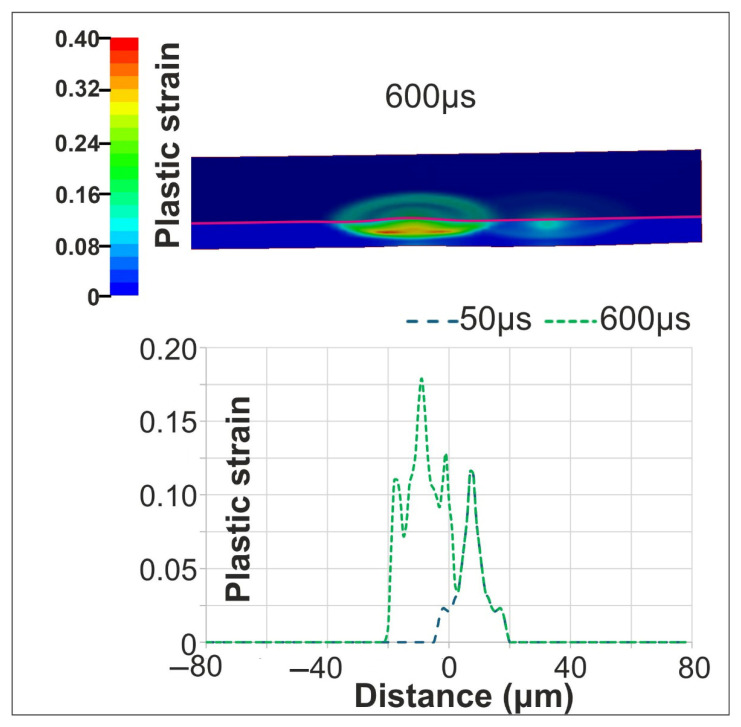
Cross-sectional plastic strain distribution along the symmetry plane at 600 μs (**top**) Evolution of the surface plastic strain following the first and second pulses along the y-axis (**bottom**).

For a PD of 2.5 μm, the first pulse produced thermomechanical behavior consistent with single-pulse irradiation, reaching a similar maximum temperature and heated depth. The second pulse, applied after a 50 μs delay, generated a more pronounced mechanical response due to the localized material modifications from the first pulse. Figure 10 depicts the plastic strain distributions at 600 μs at the middle of the target. The magenta line is used to separate the irradiated top surface of the target from the cross-section view, below that shows the inner volume. The cross section, under the magenta line, demonstrates the strain profile beneath the laser spots. At 600 μs Si is totally re-solidified and the strains presented are permanent. In contrast to all previous cases, the maximum residual plastic strain of 0.04 is located under the irradiated surface of the target, within the cross-section area presented. Furthermore, the surface plastic strain increased to 0.018, exceeding the first-pulse value of 0.012. The target required 550 μs to cool to ambient temperature following the second pulse.

**Figure 11:**
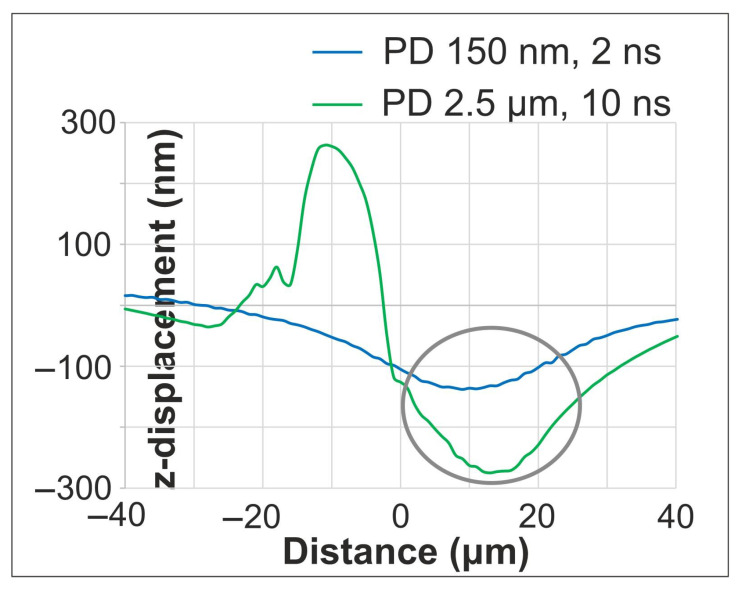
Vertical displacement profiles for lower and higher PDs at 2 ns and 10 ns, respectively, after the second pulse. A circular mark is added to highlight the ripple formation area of the graphs.

Figure 11 presents characteristic vertical displacement profiles for lower (150 nm) and higher (2.5 μm) PD at 2 ns and 10 ns, respectively, after the second pulse. At these times, ripple formation is evident within the molten region. The ripple formation closely resembles the morphology and mechanism seen in single-pulse cases (Figure 4 and Figure 6), confirming that similar fundamental process of interference patterns governs the initial stage of ripple formation. A distinct bulge is observed between −20 μm and 0 μm for the higher PD case, indicating that the thermal and mechanical influence of the second pulse persists at 10 ns. This contrasts with the lower PD case, where the material has largely solidified, demonstrating how PD governs the temporal evolution of surface dynamics. The maximum resolidified deformation depth at the end of the simulation reaches ~600 nm, which originates from hydrodynamic flow in the molten phase.

The comparison between the single- and sequential- pulse irradiation cases reveals a clear amplification of mechanical effects. In the single-pulse simulations, ripple formation originates from the interaction between the thermoelastic wavepacket launched by the laser and the waves reflected from the nearby boundary leading to standing wave patterns for shallow PDs (75–150 nm) and partial suppression at deeper PDs (~2.5 μm) due to prolonged melting and hydrodynamic flow. When a second pulse is applied after full thermal relaxation, the pre-existing residual stress and strain fields established by the leading pulse modify the mechanical state of the material and therefore amplify the deformation response, even though the absorbed energy of the second pulse is unchanged. The second pulse produces higher von Mises stresses, larger plastic strain amplitudes, and deeper surface modulation compared to the single-pulse case. This amplification effect confirms that the single-pulse analogues successfully model the mechanical boundary conditions that a leading pulse induces for a following laser pulse. The full sequential-pulse simulations then validate that in a true multi-pulse scenario, these preconditions strengthen wave interference, which enhances the persistence and regularity of LIPSS features.

## 4. Discussion

This investigation provides a robust thermomechanical framework for understanding the formation of LIPSSs on Si under ps pulsed laser irradiation. The results demonstrate that the deterministic formation of surface ripples can be governed by the excitation and interference of mechanical waves within the molten layer, a mechanism distinct from purely electromagnetic models. A central finding of this work is the identification of the PD as an important parameter for controlling the LIPSS formation pathway.

The simulations reveal that at shallow PDs (75–150 nm), energy deposition is highly superficial, creating a thin, transient molten layer. This confined volume acts as an efficient waveguide for thermoelastically generated stress waves. The interference of these waves, reflecting from intrinsic or pulse-induced boundaries, establish standing wave patterns with periodicities on the order of the laser wavelength. The brief melt duration (1–5 ns) is sufficient for this mechanical pattern to be hydrodynamically imprinted onto the molten surface but is too short for significant flow-induced smoothing, resulting in clear, persistent ripples upon re-solidification. At a deep PD (2.5 µm), the energy is deposited over a much larger volume, leading to an extensive and long-lived molten pool (~100 ns). While mechanical waves are still generated, their patterns are disrupted and smoothed out by prolonged hydrodynamic flows.

The influence of boundary geometry is also evident. When irradiation takes place near a planar boundary, the returning stress-wave components generated at the boundary interact with the continuing wavepacket from the heated region, producing a coherent interference pattern that gives rise to well-defined standing-wave modulation and surface ripples of significant amplitude. Near the vertex the superposition of doubly reflected waves reduce overall out-of-plane deformation but amplifies the transient stress oscillations, implying that the spatial confinement of the mechanical field can be exploited to modulate the ripple amplitude and uniformity.

Simulations of sequential irradiation demonstrate the cumulative nature of thermomechanical effects. When the second pulse is applied after full thermal relaxation, residual stresses and plastic strains from the first pulse act as pre-conditioning factors that enhance the mechanical response, leading to higher stress and strain amplitudes during the subsequent irradiation. This cumulative mechanical amplification leads to larger resolidified deformation depths compared to single-pulse cases. Coherent interference patterns that resemble experimentally observed LIPSSs [28] were computed.

Finally, the consistent correlation between the simulated ripple periodicities (~1.5–2 µm) and the laser wavelength (1.03 µm) emerges naturally from the acoustic properties of Si and the spatial scale of the energy deposition and confinement. This suggests that the well-known wavelength-scale periodicity of LIPSSs, often attributed solely to electromagnetic interference, can also be a direct consequence of confined mechanical wave dynamics, offering a complementary or even alternative formation pathway under specific thermal regimes.

Furthermore, the interpretation of the high stresses and resultant plastic strain observed in our simulations is supported by recent studies on laser-induced phase transitions in silicon. Under intense ultrafast laser excitation, silicon can undergo a complex, shock-driven transformation sequence (Si I → Si II → Si III/Si XII), with high-pressure polymorphs forming in both surface and bulk regions [49]. Molecular dynamics simulations further confirm that an ultrafast, reversible phase transition can be initiated at shock pressures exceeding 11 GPa [50]. While our thermomechanical model does not explicitly resolve these specific polymorphic transitions, the high von Mises stresses (1.0–1.8 GPa) and significant residual plastic strains we report are consistent with the high-pressure conditions known to drive such solid-state phase transformations. 

## 5. Conclusions

In conclusion, this study sheds light on the thermomechanical and hydrodynamic processes underlying LIPSS formation on Si wafers irradiated by ps laser pulses. It is demonstrated that the interference of thermoelastically generated stress waves, forming standing wave patterns within the molten silicon layer, is a sufficient mechanism for initiating ripple formation with laser-wavelength periodicity.

PD is identified as the key parameter dictating the outcome of laser structuring. Shallow PDs (75–150 nm) favor the formation of distinct, persistent LIPSSs by creating a thin, short-lived melt that supports stable standing waves. Deep PDs (~2.5 μm) promote hydrodynamic smoothing due to an extensive and long-lived molten pool, suppressing periodic structuring.

The geometry of wave-reflecting boundaries, whether the sample’s edges or the material modifications from previous pulses, provides a powerful means to control interference patterns and allows for the tailoring of ripple amplitude and uniformity by strategically designing the irradiation sequence and laser spot focusing.

The formation of LIPSSs is a cumulative process. Residual stress and strain from a leading pulse pre-condition the surface, mechanically amplifying the response to subsequent pulses and leading to enhanced ripple definition and amplitude, thereby explaining the pulse-overlap dependence observed experimentally.

This work significantly contributes to the deterministic laser-based surface patterning of Si by elucidating the interplay between absorption, melting, and mechanical wave dynamics. The insights gained pave the way for optimizing laser parameters (wavelength, fluence, pulse overlap) and material properties (e.g., via doping to control PD) to achieve desired surface functionalities. Future work may extend this framework by integrating electromagnetic field coupling and two-temperature models to capture the early-stage carrier dynamics, validated by experimental measurements, to further advance the deterministic control of laser-induced nanostructuring processes for Si-based photonic and microelectronic applications.

## Data Availability

The data presented in this study are openly available in ASEP depository: https://doi.org/10.57680/asep.0642329.
